# DC-Dielectrophoretic Manipulation and Isolation of Microplastic Particle-Treated Microalgae Cells in Asymmetric-Orifice-Based Microfluidic Chip

**DOI:** 10.3390/mi14010229

**Published:** 2023-01-16

**Authors:** Tianbo Gao, Kai Zhao, Jiaqi Zhang, Kaihuan Zhang

**Affiliations:** 1 Liaoning Key Laboratory of Marine Sensing and Intelligent Detection, Department of Information Science and Technology, Dalian Maritime University, Dalian 116026, China; 2 2020 X-Lab, Shanghai Institute of Microsystem and Information Technology, Chinese Academy of Sciences, Shanghai 200050, China

**Keywords:** DC-dielectrophoretic manipulation, *Chlorella* microalgae cells, microplastic particles, asymmetric orifice structure

## Abstract

A novel direct-current dielectrophoretic (DC–DEP) method is proposed for the manipulation and isolation of microplastic particle (MP)-treated microalgae cells according to their dielectric properties in a microfluidic chip. The lateral migration and trajectory of the microalgae cells were investigated. To induce stronger DC–DEP effects, a non-homogeneous electric-field gradient was generated by applying the DC electric voltages through triple pairs of asymmetric orifices with three small orifices and one large orifice located on the opposite microchannel wall across the whole channel, leading to the enhanced magnitude of the non-uniform electric-field gradient and effective dielectrophoretic area. The effects of the applied voltage, the polystyrene (PS) adsorption coverage, and thickness on the DC–DEP behaviors and migration were numerically investigated, and it was found that the effect of the PS adsorption thickness of the Chlorella cells on the DC–DEP behaviors can be neglected, but the effect on their trajectory shifts cannot. In this way, the separation of 3 µm and 6 µm Chlorella coated with 100% PS particles and the isolation of the Chlorella cells from those coated with various coverages and thicknesses of PS particles was successfully achieved, providing a promising method for the isolation of microalgae cells and the removal of undesired cells from a target suspension.

## 1. Introduction

Microalgae are widely distributed around the world, and they are rich in protein, biological antibiotics, and oil [1,2], which are widely utilized in various fields, such as biotechnology, the food industry, and environmental protection [3,4,5]. As microalgae show outstanding biological properties, including high photosynthetic efficiency and simple structure, they can grow well under extreme environmental conditions of heavy metals, high salinity, nutrient deficiencies, and extreme temperatures. Moreover, microalgae show high potential for use in microbial remediation techniques for microplastic particles due to their high affinity, abundant binding sites, and large specific surface area [6,7,8,9,10]. In addition to their excellent removal capacity and eco-friendly properties, microalgae-removal technologies show advantages, including stable and simple processes, non-toxic side effects, fast growth rates compared to higher plants, and the possibility of producing value-added products, such as biofuels [11,12]. Therefore, the isolation of microalgae from contaminated objects (such as microplastics or impurities) is crucial. To address this challenge, the microfluidic system becomes an opportunity for the manipulation of microscale and nanoscale targets [13]. Compared with various sorting methods, dielectrophoresis is one of the most reliable techniques to provide the accurate manipulation of microalgae and controllable dielectrophoretic effects on the targets due to the advantages of label-free sorting, low sample consumption, and tunability to analyze selectively and sensitively [14,15,16,17,18,19,20].

Dielectrophoresis refers to the movement of polarized particles suspended in a dielectric solution due to the polarization difference between the particles and the suspension in a non-homogeneous electric field. Generally, a non-homogeneous electric-field gradient can be induced by applying the alternating current (AC) electric fields through the embedded micro-electrode array with patterned geometry and structure inside the microchannel [16,21,22,23,24] or the direct current (DC) electric field via the external electrodes over arrays of patterns of insulating obstacles or hurdles across the whole channel [25,26,27,28,29]. When applying high-electricity fields, the particle may exhibit a nonlinear electrophoresis effect [30,31]. Since the magnitude of the dielectrophoretic forces and the direction of the dielectrophoretic behaviors are determined by the size and dielectric properties of the particles, the selective and sensitive manipulation of particles is enabled by using the DEP methods. The electrode-based AC–DEP systems can induce a strong non-homogeneous electric-field gradient, but they involve complicated microelectrode fabrication, electrode fouling, and electrochemical reactions over the electrode surface. These shortcomings are overcome in the insulator-based DC-DEP systems. However, to generate stronger DC-DEP effects, high voltage is necessary which may induce Joule heating effects.

In this work, a novel asymmetric-orifice-based DC–DEP microfluidic chip is designed for the manipulation and isolation of *Chlorella* and *Chlorella* adsorbed and coated with polystyrene microplastic particles. By applying a relatively low DC electric field via the asymmetric orifice structures, i.e., a triple small orifice and a large orifice on the opposite microchannel wall, through the whole channel, the aforementioned effects, such as the complex fabrication of inserted microelectrodes, electrode fouling and electrochemical reaction, and Joule heating, are avoided and a locally strong inhomogeneous electric field is induced, resulting in the enlarged magnitude of the DC–DEP forces and the corresponding effective area. In this way, stronger DC–DEP effects on the *Chlorella* cells lead to larger migration shifts. To better understand the DC–DEP trajectory of the *Chlorella* cells adsorbed and coated with microplastic particles, the distribution of the electric-field gradient and the Clausius–Mossotti factors as a function of the electrical conductivity of the surrounding medium were studied. Moreover, the effects of the applied voltage, the PS adsorption coverage, and thickness on their DC–DEP behaviors and migration were investigated. It was found that the effect of the PS adsorption thickness of the *Chlorella* cells on the DC–DEP behaviors can be neglected, but not the effect on their trajectory shifts. In addition, the separation of 3 µm and 6 µm *Chlorella* coated with 100% PS particles and the isolation of the *Chlorella* cells from those coated with various coverages and thicknesses of PS particles was successfully achieved.

## 2. Dielectrophoresis

Dielectrophoresis refers to the movement of polarizable particles suspended in a non-homogeneous electric field. The dielectrophoretic force exerted on a spherical particle is generally expressed as [32].

(1) FDEP=2πεm r3 Re(fCM)(∇|E|2)fCM=(εp*−εm*εp*+2εm*)

where r represents the particle radius, $\varepsilon_{p}^{*}$ and $\varepsilon_{m}^{*}$ are the complex permittivity of the particle and surrounding medium, respectively, $\varepsilon^{*}=\varepsilon -\left(j\sigma /\omega \right)$, $\nabla {\left|E\right|}^{2}$ describes the electric-field gradient, the electrical conductivity and the angular frequency of electrical field are given by σ and ω, $j=\sqrt{-1}$, and the real part of Clausius–Mossotti (CM) factor Re(fCM) demonstrates the polarizability between the particle and the suspension. For the homogeneous ellipsoidal particles, the DEP forces still scale linearly with the particle size and the electric-field gradient, and the directions of the DEP behaviors are determined by the Re(fCM), although they experience a frequency-dominant alignment torque and the principal axe tends to align with the electric field [33].

**Figure 1:**
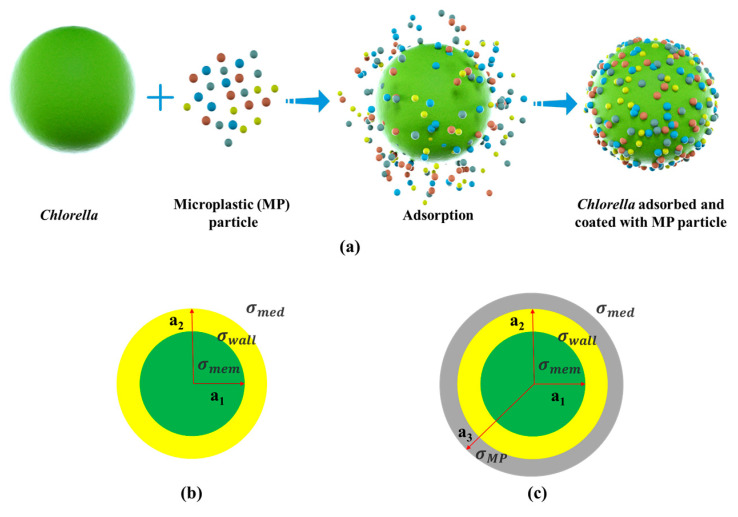
Schematic illustration of (**a**) the *Chlorella* adsorbed with microplastic (MP) particles, (**b**) the single-shell structure model of *Chlorella*, (**c**) the double-shell structure model of *Chlorella* adsorbed and coated with a layer of MP particles.

The adsorption process for the *Chlorella* coated with microplastic (MP) particles and the *Chlorella* coated with a MP-particle layer are shown in Figure 1. The manipulation of the *Chlorella* by the dielectrophoretic effects come from the combination of the dielectrophoretic forces exerted on the uncoated *Chlorella* and the MP-coated *Chlorella* and the total dielectrophoretic forces is described as [34]:

(2) $$F_{DEP,\;eff}=F_{DEP,\;\;C}+F_{DEP,C-MP1}+F_{DEP,C-MP2}+\ldots +F_{DEP,C-MPn}$$

The total volume of the *Chlorella* sphere and the *Chlorella* sphere coated with MP particles is approximately equal to the original *Chlorella*, which is written as [34]

(3) $$\begin{array}{cc} V & =\frac{4}{3}\pi a^{3}=V_{eff,\;C}+V_{eff,\;C-MP1}+V_{eff,\;C-MP2}+\ldots +V_{eff,\;C-MPn}\\ & =\frac{4}{3}\pi a_{eff,C}^{3}+\frac{4}{3}\pi a_{eff,C-MP1}^{3}+\frac{4}{3}\pi a_{eff,C-MP2}^{3}+\ldots +\frac{4}{3}\pi a_{eff,C-MPn}^{3} \end{array}$$

(4) $$\begin{array}{cc} V_{eff,\;C-MP1}=C_{1}*V, & V_{eff,\;C-MP2}=C_{2}*V,\;\ldots ,\;V_{eff,\;C-MPn}=C_{n}*V\\ V_{eff,\;C}=V- & V_{eff,\;C-MP1}-V_{eff,\;C-MP2}-\ldots -V_{eff,\;C-MPn}\\ & =\left(1-C_{1}-C_{2}-\ldots -C_{n}\right)*V \end{array}$$

(5) $$\begin{array}{cc} a_{eff,C-MP1}^{3}= & C_{1}*a^{3},\;a_{eff,C-MP2}^{3}=C_{2}*a^{3},\;\ldots ,\;a_{eff,C-MPn}^{3}=C_{n}*a^{3}\\ a_{eff,C}^{3}= & a^{3}-a_{eff,C-MP1}^{3}-a_{eff,C-MP2}^{3}-\ldots -a_{eff,C-MPn}^{3}\\ & =\left(1-C_{1}-C_{2}-\ldots -C_{n}\right)*a^{3} \end{array}$$

where a represents the radius of the original *Chlorella*, and $a_{eff,C}$ and $a_{eff,C-MPn}$ describe the effective radius of the *Chlorella* sphere and the *Chlorella* sphere coated with MP particles, respectively. The *C_n_* is the volumetric coverage of the coated MP particles, and (1 − *C_n_*) is the part of the *Chlorella* sphere with no coatings. The n describes different kinds of MP particles. The surface area for the coated-MP-particle part can be expressed as

(6) $$VS_{C-MPn}=P_{n}*4\pi a^{2}=2\pi ah$$

where *P_n_* represents the MP-particle-coating coverage on the *Chlorella* sphere and $h=2aP_{n}$. The corresponding volume of the coated area of the *Chlorella* sphere and the volumetric coverage *C_n_* can be written as

(7) $$\begin{array}{c} V_{eff,\;C-MPn}=\pi h^{2}\left(a-\frac{h}{3}\right)=P_{n}^{2}\times 4\pi a^{3}\left(1-\frac{2P_{n}}{3}\right)\\ C_{n}=\frac{V_{eff,\;C-MPn}}{V}=\frac{P_{n}^{2}\times 4\pi a^{3}\left(1-\frac{2P_{n}}{3}\right)}{\frac{4\pi a^{3}}{3}}=3P_{n}^{2}\left(1-\frac{2P_{n}}{3}\right) \end{array}$$

Therefore, the volumetric coverage of the coated MP particles on the *Chlorella* sphere *C_n_*, and the effective radius of the *Chlorella* sphere $a_{eff,C}$ and the *Chlorella* sphere coated with MP particles $a_{eff,C-MPn}$ can be obtained given the MP-particle-coating coverage on the *Chlorella* sphere *P_n_*. Hence, the total DEP forces exerted on the *Chlorella* are described as

(8) $$\begin{array}{cc} F_{DEP,\;eff} & =F_{DEP,\;\;C}+F_{DEP,C-MP1}+F_{DEP,C-MP2}+\ldots +F_{DEP,C-MPn}\\ =2\pi \varepsilon_{m}\;[a_{eff,C}^{3}* & Re\left(f_{CM,C}\right)+a_{eff,C-MP1}^{3}*Re\left(f_{CM,C-MP1}\right)+a_{eff,C-MP2}^{3}\\ & *Re\left(f_{CM,C-MP2}\right)+\ldots +a_{eff,C-MPn}^{3}*Re\left(f_{CM,C-MPn}\right)]\left(\nabla {\left|E\right|}^{2}\right)\\ =2\pi \varepsilon_{m}\;a^{3} & [\left(1-C_{1}-C_{2}-\ldots -C_{n}\right)*Re\left(f_{CM,C}\right)+C_{1}*Re\left(f_{CM,C-MP1}\right)+C_{2}\\ & *Re\left(f_{CM,C-MP2}\right)+\ldots +C_{n}*Re\left(f_{CM,C-MPn}\right)](\nabla {\left|E\right|}^{2}) \end{array}$$

where the $f_{CM,C}$ for the *Chlorella* is expressed as

(9) $$\begin{array}{c} Re\left(f_{CM,\;C}\right)=\left(1-C_{1}-C_{2}-\ldots -C_{n}\right)*Re\left(f_{CM,C}\right)+C_{1}*Re\left(f_{CM,C-MP1}\right)+C_{2}*\\ Re\left(f_{CM,C-MP2}\right)+\ldots +C_{n}*Re\left(f_{CM,C-MPn}\right) \end{array}$$

Based on the shell model [33] of CM factor the *Chlorella*, the complex permittivity for the *Chlorella* in Equation (1) is given by

(10) $$\varepsilon_{C}^{*}=\varepsilon_{wall}^{*}\left[\gamma_{12}^{3}+2\left(\frac{\varepsilon_{mem}^{*}-\varepsilon_{wall}^{*}}{\varepsilon_{mem}^{*}+2\varepsilon_{wall}^{*}}\right)\right]/\left[\gamma_{12}^{3}-\left(\frac{\varepsilon_{mem}^{*}-\varepsilon_{wall}^{*}}{\varepsilon_{mem}^{*}+2\varepsilon_{wall}^{*}}\right)\right]$$

where the factor $\gamma_{12}=a/a_{1}$, a and $a_{1}$ represent the radius of the *Chlorella* and its membrane, respectively. The complex permittivity for the *Chlorella* sphere coated with MP particles is expressed as

(11) $$\varepsilon_{C-MPn}^{*}=\varepsilon_{MPn}^{*}\left[\gamma_{23}^{3}+2\left(\frac{\varepsilon_{C}^{*}-\varepsilon_{MPn}^{*}}{\varepsilon_{C}^{*}+2\varepsilon_{MPn}^{*}}\right)\right]/\left[\gamma_{23}^{3}-\left(\frac{\varepsilon_{C}^{*}-\varepsilon_{MPn}^{*}}{\varepsilon_{C}^{*}+2\varepsilon_{MPn}^{*}}\right)\right]$$

where the factor $\gamma_{23}=a_{2}/a$, $a_{2}$ is the radius of the *Chlorella* sphere with the MP-particle-coating layer. When only applying the DC electric field, the $f_{CM,C}$ becomes solely dependent on the electrical conductivity [35], which is rewritten by

(12) $$\begin{array}{c} Re\left(f_{CM,\;C}\right)=\left(1-C_{1}-C_{2}-\ldots -C_{n}\right)*\frac{\sigma_{C}-\sigma_{m}}{\sigma_{C}+2\sigma_{m}}+C_{1}*\frac{\sigma_{C-MP1}-\sigma_{m}}{\sigma_{C-MP1}+2\sigma_{m}}+C_{2}*\\ \frac{\sigma_{C-MP2}-\sigma_{m}}{\sigma_{C-MP2}+2\sigma_{m}}+\ldots +C_{n}*\frac{\sigma_{C-MPn}-\sigma_{m}}{\sigma_{C-MPn}+2\sigma_{m}}\\ \sigma_{C-MPn}=\sigma_{MPn}\left[\gamma_{23}^{3}+2\left(\frac{\sigma_{C}-\sigma_{MPn}}{\sigma_{C}+2\sigma_{MPn}}\right)\right]/\left[\gamma_{23}^{3}-\left(\frac{\sigma_{C}-\sigma_{MPn}}{\sigma_{C}+2\sigma_{MPn}}\right)\right] \end{array}$$

where $\sigma_{C}$ is the electrical conductivity of the *Chlorella* sphere. The DC–DEP force exerted on the *Chlorella* coated with MP particles can be described as

(13) $$\begin{array}{cc} F_{DEP,\;eff}=2\pi \varepsilon_{m}\; & a^{3}[\left(1-C_{1}-C_{2}-\ldots -C_{n}\right)*\frac{\sigma_{C}-\sigma_{m}}{\sigma_{C}+2\sigma_{m}}+C_{1}*\frac{\sigma_{C-MP1}-\sigma_{m}}{\sigma_{C-MP1}+2\sigma_{m}}\\ & +C_{2}*\frac{\sigma_{C-MP2}-\sigma_{m}}{\sigma_{C-MP2}+2\sigma_{m}}+\ldots +C_{n}*\frac{\sigma_{C-MPn}-\sigma_{m}}{\sigma_{C-MPn}+2\sigma_{m}}](\nabla {\left|E\right|}^{2}) \end{array}$$

## 3. Numerical Methods

**Figure 2:**
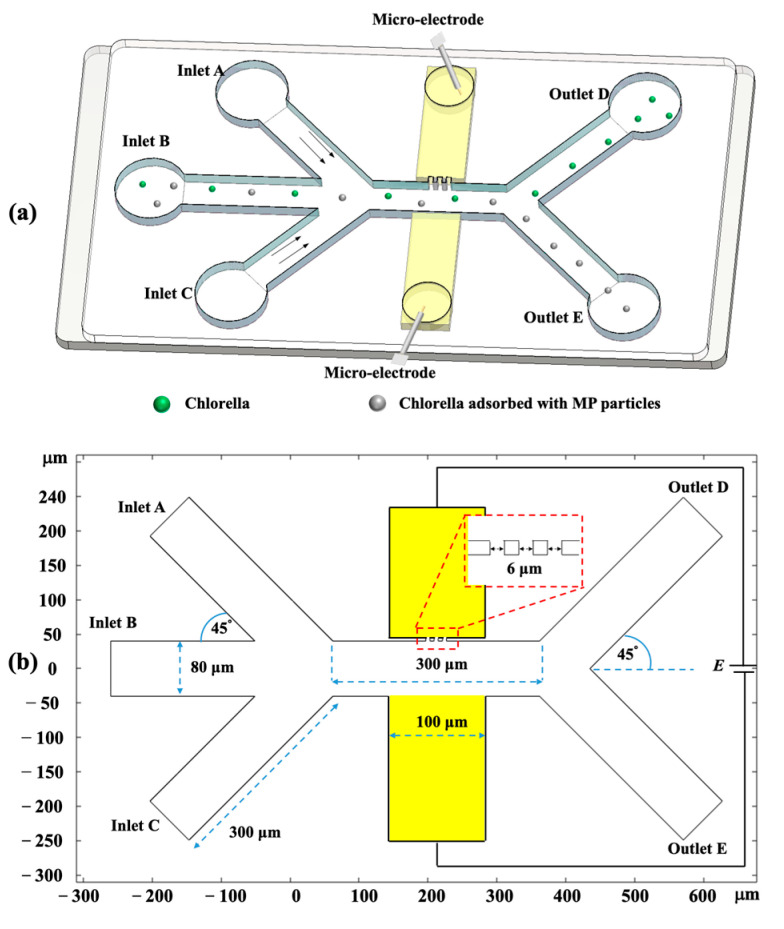
Schematic illustration of (**a**) side view and (**b**) top view of the asymmetric-orifice-based DC–DEP microfluidic chip with triple-pairs-of-orifices structure.

It can be inferred from Equation (13) that the magnitude of the total DC–DEP forces scales linearly with the diameter of *Chlorella* sphere and the non-homogeneous electric-field gradient, while the direction of the dielectrophoretic trajectory is dependent on the sign of *Chlorella*-dependent $Re\left(f_{CM,\;C}\right)$. In this way, the size-based sorting of *Chlorella* is straightforward. In addition, as discussed in Equation (8), the value of $Re\left(f_{CM,\;C}\right)$ depends on the coverage, thickness, and type of MP-particle coating. If the $Re\left(f_{CM,\;C}\right)>0$, the *Chlorella* is attracted towards the maximum electric-field gradient by the positive DEP forces. By contrast, they are repelled away by negative DEP effects and move away from the high-electric-field area. In this way, the isolation of *Chlorella* with different MP-particle coatings can be achieved. In this study, a novel DC–DEP manipulation method for the isolation of *Chlorella* microalgae cells is proposed by applying a relatively low electric field via the triple-orifice-based asymmetric-orifice structure across the whole microchannel, which is shown in Figure 2. This channel consists of three inlet and two outlet channels with a width of 80 μm, connected by a horizontal mainchannel, which is 300 μm in length. All these channels are shifted 45° from the horizontal channel. In this mainchannel, the asymmetric orifices with a triple small orifice are 6 μm wide and a large orifice with a width of 100 μm is perpendicular to the mainchannel. The electric field is applied through the embedded micro-electrodes to induce the inhomogeneous electric field in the asymmetric orifice region. When passing through the non-uniform electric-field gradient, the *Chlorella* microalgae cells undergo various dielectrophoretic forces to show different migration behaviors and flow into individual outlets.

In this work, the following equations and boundary conditions were calculated by utilizing COMSOL 5.4 software, and a numerical investigation into the manipulation and isolation of MP-treated *Chlorella* microalgae cells in asymmetric-orifice-based microfluidic chip by using DC–DEP was conducted. To induce the non-homogeneous electric-field gradient, the electric field was solved in a stationary solver. To visualize the migration of these microalgae cells, the particle-tracing module was applied to track their trajectories, varying with different DEP effects. To better understand the DC–DEP manipulation of the *Chlorella* microalgae cells, extensive numerical simulations on the effect of applied electrical voltage, and the PS adsorption coverage and thickness, were analyzed.

### 3.1. Electric Field

The distribution of the electric filed is governed by the Laplace’s equation

(14) $$\nabla^{2}\varphi =0$$

(15) $$\begin{array}{c} \varphi =V_{1}\;\mathrm{at}\;\mathrm{the}\;\mathrm{small}\;\mathrm{orifice}\\ \varphi =0\;V\;\mathrm{at}\;\mathrm{the}\;\mathrm{large}\;\mathrm{orifice}\\ \;\hat{n}\left(\nabla \varphi \right)=0\;\mathrm{at}\;\mathrm{channel}\;\mathrm{walls} \end{array}$$

where the electric potential is applied at the inserted micro-electrode pads and $\;\hat{n}$ represents the unit normal vector.

### 3.2. Flow Field

The liquid flow inside the microfluidic chip is considered as incompressible laminar flow and governed by Navier–Stokes equation

(16) $$\begin{array}{c} \rho \left[\frac{\partial \vec{u}}{\partial t}+\vec{u}\cdot \nabla \vec{u}\right]=-\nabla P+\mu \nabla^{2}\vec{u}\\ \nabla \cdot \vec{u}=0 \end{array}$$

where Equation (16) illustrates the continuity equation and the term of $\frac{\partial \vec{u}}{\partial t}$ in Equation (16) is neglected as it is the steady flow. Furthermore, the inertia $\vec{u}\cdot \nabla \vec{u}$ is also negligible as the liquid is moving at a relatively low velocity in the microchannel. The velocity is described by $\vec{u}$, the density and viscosity of the solution are ρ and μ, respectively, and ∇P demonstrates the gradient of the pressure.

The non-slip boundary condition at the microchannel walls is described by Equation (17). The inlets and outlet of the microchannels are set with specific velocity values.

(17) $$\begin{array}{c} u=u_{1}\;\mathrm{at}\;\mathrm{the}\;\mathrm{inlets}\\ P=0\;\;\mathrm{at}\;\mathrm{the}\;\mathrm{outlets}\\ \vec{u}=0\;\mathrm{at}\;\mathrm{channel}\;\mathrm{walls} \end{array}$$

### 3.3. Particle Tracing

To visualize the migration of the *Chlorella* in the asymmetric-orifice-based DC–DEP microchannel, the trajectories are calculated and coupled with the applied electric field and flow field, whose movement is governed by Newton’s second law [36,37]

(18) $${\vec{F}}_{t}=m_{p}\frac{d\vec{v}}{dt}$$

where the net force, including the applied dielectrophoretic force and the drag force, is represented by ${\vec{F}}_{t}$, and the mass and velocity of the moving *Chlorella* are described by $m_{p}$ and $\vec{v}$, respectively.

The dielectrophoretic force acting on the *Chlorella* adsorbed with PS microplastic particles is described as

(19) $$\begin{array}{cc} F_{DEP,\;eff}=2\pi \varepsilon_{m}\; & a^{3}[\left(1-C_{1}-C_{2}-\ldots -C_{n}\right)*\frac{\sigma_{C}-\sigma_{m}}{\sigma_{C}+2\sigma_{m}}+C_{1}*\frac{\sigma_{C-MP1}-\sigma_{m}}{\sigma_{C-MP1}+2\sigma_{m}}\\ & +C_{2}*\frac{\sigma_{C-MP2}-\sigma_{m}}{\sigma_{C-MP2}+2\sigma_{m}}+\ldots +C_{n}*\frac{\sigma_{C-MPn}-\sigma_{m}}{\sigma_{C-MPn}+2\sigma_{m}}]\left(\nabla {\left|E\right|}^{2}\right) \end{array}$$

where $C_{n}=3P_{n}^{2}\left(1-\frac{2P_{n}}{3}\right)$ and *P_n_* represents the coating coverage.

The drag force exerted on the *Chlorella* in the laminar flow is given by

(20) $$\begin{array}{c} {\vec{F}}_{drag}=\frac{1}{\tau_{p}}m_{p}\left(\vec{u}-\vec{v}\right)\\ \tau_{p}=\frac{2\rho_{p}r^{2}}{9\mu } \end{array}$$

where the density and radius of the *Chlorella* are demonstrated by $\rho_{p}$ and r, respectively, and the μ represents the dynamic viscosity of the fluid. All the parameters utilized in the numerical study are shown in Table 1.

## 4. Discussion

### 4.1. Simulation of the Electric Field

**Figure 3:**
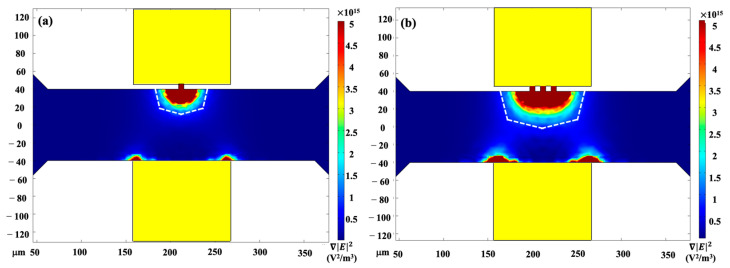
The numerical distribution of the electric field in the asymmetric-orifice-based microfluidic chip: (**a**) one pair and (**b**) triple pairs of asymmetric orifices.

The numerical simulation of the distribution of the non-homogeneous electric field across the asymmetric orifice was conducted by using COMSOL 5.4. By applying the electric field across the structure of the orifices via the embedded micro-electrode pads, the electric-field gradient is generated, where the small orifice has the strongest non-uniform electric fields. As shown in Figure 3a, the dark-red semicircle, i.e., the non-homogeneous electric-field gradient, is approximately 25 μm in radius. It can be inferred from Equation (1) that the stronger electric field gradient, i.e., the magnitude of the DEP effects, leads to a higher sorting efficiency and resolution. By structuring the asymmetric orifices’ geometry to triple pairs, the semicircle is increased to approximately 40 μm (shown in Figure 3b), resulting in an enlarged region of non-homogeneous electric-field gradient and an extension of the time period of the DEP’s effects on the targets. Subsequently, the *Chlorella* adsorbed with PS particles experiences DEP forces, and their trajectory migrations change when passing over this area.

In addition, in the proposed triple-asymmetric-orifice-based DC–DEP microfluidic chip, the $\nabla {\left|E\right|}_{max}^{2}$ calculated is in the order of 10^15^ V^2^/m^3^ with 5 V employed via the asymmetric orifices over 40 μm between the microelectrodes. Nevertheless, the value of the $\nabla {\left|E\right|}_{max}^{2}$ is approximately ~10^11^–10^15^ V^2^/m^3^ with 70–1200 V/cm applied through the whole microchannel in traditional insulator-based DC–DEP devices. Therefore, the application of a relatively low electrical voltage across the asymmetric-orifice-based microchannel can induce a sufficiently strong non-homogeneous electric-field gradient and prevent the potential Joule heating effect. This allows stronger DEP forces on the particles, enabling the manipulation of smaller particles with higher accuracy and resolution.

### 4.2. Effect of the Applied Voltage

**Figure 4:**
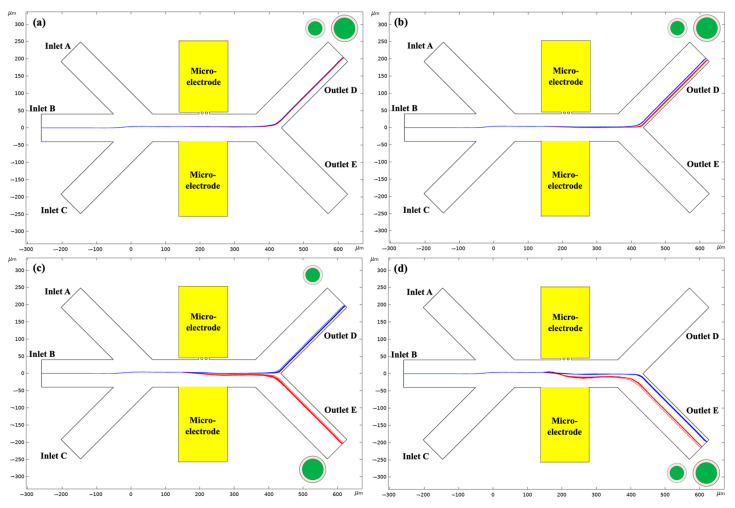
The migration of 3-micrometer and 6-micrometer *Chlorella* adsorbed and coated with 100% PS particles varying with the applied electric voltage: (**a**) 5 V, (**b**) 15 V, (**c**) 25 V, (**d**) 40 V. The thickness of the coated layer, i.e., the diameter of the PS particles, is 0.6 µm and the electrical conductivity of the surrounding solution is $\sigma_{m}$= 1 × 10^−7^ S/m.

To examine the effect of the non-homogeneous electric field gradient on the migration of 3-micrometer and 6-micrometer *Chlorella* adsorbed and coated with 100% PS particles, different electrical voltages were applied at the embedded micro-electrodes through the asymmetric orifices from 5 V to 40 V. In this study, the thickness of the coated layer, i.e., the diameter of the PS particles, is 0.6 µm and the electrical conductivity of the surrounding solution is $\sigma_{m}$= 1 × 10^−7^ S/m. The separation of the 3-micrometer and 6-micrometer *Chlorella* coated with 100% PS particles varying with the applied non-uniform electric field is shown in Figure 4.

**Figure 5:**
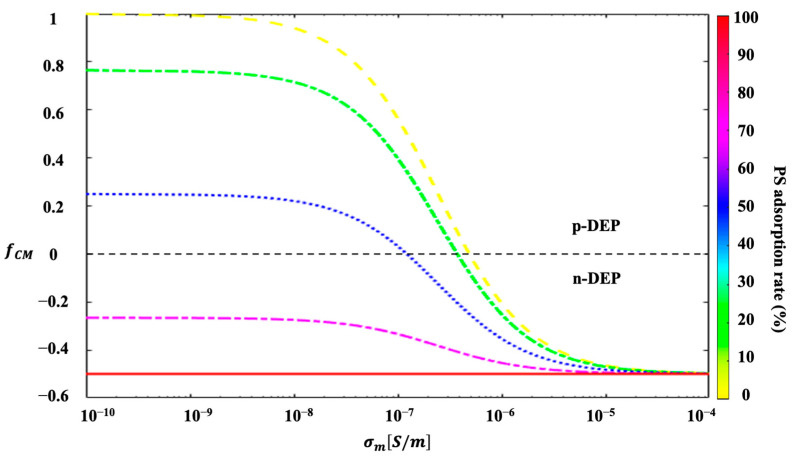
The CM factor of 3-micrometer *Chlorella* coated with various different coverage levels of PS particles, which varied with the electrical conductivity of the surrounding medium. The thickness of the PS-particle coating was 0.6 µm.

As indicated in Figure 5, for the *Chlorella* coated with 100% PS particles, the $\left(f_{CM,\;C}\right)<0$ and they experienced negative DEP effects. It can be inferred from Equation (13) that the magnitude of the total DC–DEP forces linearly scaled with the diameter of the *Chlorella* sphere and the non-homogeneous electric-field gradient, leading to increases in the value of the DC–DEP forces with the applied electrical voltages. Therefore, the magnitude of the DC–DEP force exerted on the 6-micrometer was approximately eight times stronger than that on the 3-micrometer *Chlorella*, resulting in different trajectory migrations. As shown in Figure 4a, when the applied voltage was 5 V, the mixed *Chlorella* cells flowed together into the outlet D as the migration shifts were small between the large *Chlorella* and the small *Chlorella* due to the relatively weak DC–DEP effects. When increasing the voltage to 15 V, the trajectory migrations for the mixture were enlarged but not large enough; subsequently, they continued moving into outlet E. A sufficient level of migration was induced for the mixed large and small *Chlorella* after they passed through the asymmetric orifices when the applied voltage was 25 volts, and they moved into the individual outlets, D and E, respectively. Therefore, the sorting of the 3-micrometer and 6-micrometer *Chlorella* adsorbed and coated with 100% PS particles was achieved. However, when further increasing the applied voltage to 45 V, the large *Chlorella* and the small *Chlorella* moved into outlet E together due to the overly strong DC–DEP effects, and the separation could not be achieved.

### 4.3. Effect of the PS Adsorption Coverage

**Figure 6:**
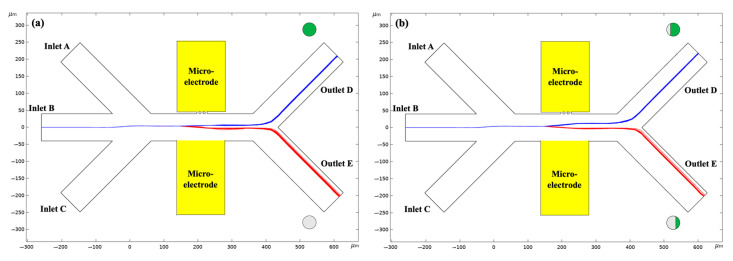
(**a**) The isolation of 3-micrometer *Chlorella* from the *Chlorella* fully coated with PS microplastic particles. (**b**) The separation of 3-micrometer *Chlorella* coated with 25% and 75% PS particles. The applied voltages were 40 and 45 volts, respectively. The thickness of the PS-particle coating was 0.6 μm and the electrical conductivity of medium used in this study was $\sigma_{m}=$ 1 × 10^−7^ S/m.

The value of 3-micrometer *Chlorella* coated with five different coverages of PS microplastic particles, i.e., 0%, 25%, 50%, 75%, and 100%, as a function of the electrical conductivity of the surrounding solution, is shown in Figure 5. The thickness of the adsorbed PS particles (the diameter of the PS particle) was 0.6 µm. Due to the selection of the specific electrical conductivity of the suspension, the *Chlorella* with different coating coverages of PS particles demonstrate opposite DEP behaviors, i.e., positive and negative DEP effects, respectively. In this way, the separation of the 3-micrometer *Chlorella* coated with 25% and 75% PS particles and the isolation of the 3-micrometer *Chlorella* from the *Chlorella* fully coated with PS particles experiencing opposite DC–DEP forces, where the selected electrical conductivity of the medium is $\sigma_{m}=$ 1 × 10^−7^ S/m, are shown in Figure 6.

As discussed in Equation (8), the value of $Re\left(f_{CM,\;C}\right)$ depends on the MP-particle-coating coverage. If the $Re\left(f_{CM,\;C}\right)>0$, the *Chlorella* are attracted towards the maximum electric-field gradient by the positive DEP forces. By contrast, they are repelled away by negative DEP effects and move away from the high-electric -field area. When the mixture of the 3-micrometer *Chlorella* and the *Chlorella* fully coated with PS microplastic particles moved through the small-orifice region, which had the strongest non-homogeneous electric-field gradient (shown in Figure 6a), they demonstrated opposite DC–DEP behaviors, i.e., $Re\left(f_{CM,\;C}\right)\approx$ 0.6 for the *Chlorella*, and they continued to flow into the outlet D after undergoing positive DC–DEP effects, while $\left(f_{CM,\;C}\right)\approx -$0.5 for the *Chlorella* fully coated with PS microplastic particles, and they were repelled away from the small orifices by the negative DC–DEP forces and moved into outlet E. Moreover, the mixed *Chlorella* coated with 25% and 75% PS particles were well separated into outlets D and E when experiencing the positive ($Re\left(f_{CM,\;C}\right)\approx$ 0.4) and negative ($\left(f_{CM,\;C}\right)\approx -$0.3) DC–DEP forces, respectively (as shown in Figure 6b).

### 4.4. Effect of the PS Adsorption Thickness

**Figure 7:**
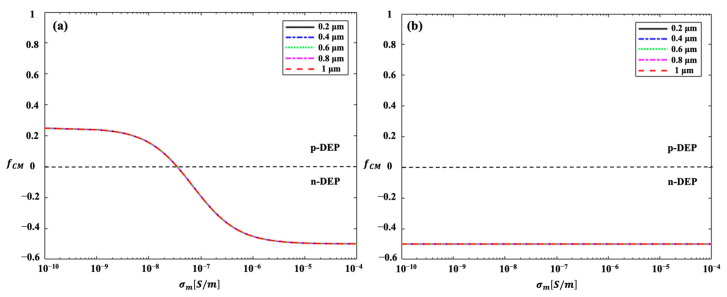
The CM factor of 3-micrometer *Chlorella* coated with (**a**) 50% and (**b**) 100% PS particles coverage but with various different PS-particle-coating thicknesses, i.e., diameters of PS particles of 0.2 µm, 0.4 µm, 0.6 µm, 0.8 µm, and 1 µm, varying with the electrical conductivity of the surrounding medium.

The CM factor of the 3-micrometer *Chlorella* coated with 50% and 100% PS-particle coverage but various different PS-particle-coating thicknesses, i.e., diameters of PS particles of 0.2 µm, 0.4 µm, 0.6 µm, 0.8 µm, and 1 µm, varying with the electrical conductivity of the surrounding medium, is shown in Figure 7.

**Figure 8:**
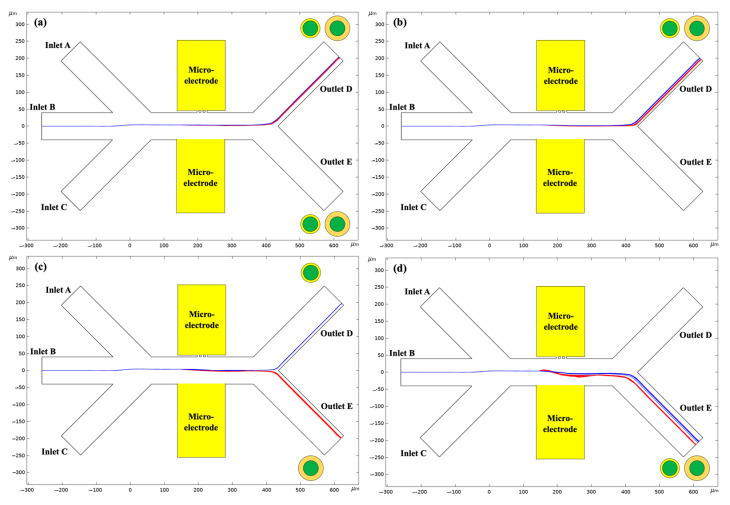
The migration of 3-micrometer *Chlorella* coated with 100% PS particles but two different PS-particle-coating thicknesses of 0.2 µm and 1 µm, respectively, varying with the applied electric voltage: (**a**) 10 V, (**b**) 20 V, (**c**) 30 V, (**d**) 50 V. The electrical conductivity of the medium used in this study was $\sigma_{m}$= 1 × 10^−7^ S/m.

It can be inferred from Figure 7 that the CM factors for the *Chlorella* with different PS-particle-coating coverages as a function of the electrical conductivity of the suspension were different, but were essentially identical with different coating thicknesses, i.e., diameters of PS particles. Therefore, *Chlorella* with different coating thicknesses always display the same DC–DEP behaviors when the given electrical conductivity of the surrounding solution is applied, resulting in a negligible effect of the PS-particle-coating thickness, i.e., the diameters of the PS particles, on their DC–DEP migrations. In this way, the separation of 3-micrometer *Chlorella* coated with 100% PS-particle coverage but with two different PS-particle coating thicknesses, of 0.2 µm and 1 µm, respectively, as a function of the electric voltage applied with the negative DC–DEP forces, is shown in Figure 8. As discussed above, the magnitude of the DC–DEP forces was proportional to the diameters of the *Chlorella* cells; the DC–DEP effects on the *Chlorella* with a coating thickness of 1 µm, i.e., with a larger effective diameter, was stronger than that on the *Chlorella* with the coating thickness of 0.2 µm, i.e., smaller effective diameter, leading to the size-based sorting. As shown in Figure 8c, the successful separation of the *Chlorella* coated with the 100% PS-particle coverage but two different PS-particle-coating thicknesses, of 0.2 µm and 1 µm, was achieved.

## 5. Conclusions

The dielectrophoretic manipulation and isolation of microplastic-particles-based *Chlorella* microalgae cells under a DC electric field in a triple-asymmetric-orifice-based microfluidic chip was numerically conducted. To generate stronger DC–DEP effects, a non-homogeneous electric-field gradient was induced by applying the DC electric voltages through triple pairs of asymmetric orifices with three small orifices and one large orifice located on the opposite microchannel wall across the whole channel. In this way, the magnitudes of the non-uniform electric-field gradient and the corresponding effective region were enhanced, leading to stronger DC–DEP forces and larger migration shifts for the *Chlorella* cells. To better understand the DC–DEP trajectory of the *Chlorella* cells adsorbed and coated with microplastic particles, the distribution of the electric-field gradient and the Clausius–Mossotti factors as a function of the electrical conductivity of the surrounding medium were studied. Moreover, the effects of the applied voltage, the PS adsorption coverage and thickness on their DC–DEP behaviors, and migration were numerically investigated. The effect of the PS adsorption thickness of the *Chlorella* cells on the DC–DEP behaviors can be neglected, but the effect on their trajectory shifts cannot. In addition, the separation of 3-micrometer and 6-micrometer *Chlorella* coated with 100% PS particles and the isolation of the *Chlorella* cells from those coated with various coverages and thicknesses of PS particles was successfully achieved. Therefore, the ability to separate mixed *Chlorella* microalgae-cell populations by adjusting the electrical conductivity of the suspension on the chip identifies the proposed method as a promising technique through which to precisely select *Chlorella* microalgae cells in complex microalgae-cell populations and remove undesired cells from a target suspension.

## Figures and Tables

**Table 1:** Values of parameters utilized in the numerical study.

Parameters	Values
$\mathrm{Dielectric}\;\mathrm{constant}\;\mathrm{of}\;\mathrm{water},\;\varepsilon_{w}$	80
$\mathrm{Permittivity}\;\mathrm{of}\;\mathrm{vacuum},\;\varepsilon_{0}$ (F/m)	8.85 × 10^−12^
$\mathrm{Density}\;\mathrm{of}\;\mathrm{water},\;\rho_{w}$ (kg/m^3^)	1000
$\mathrm{Dynamic}\;\mathrm{viscosity}\;\mathrm{of}\;\mathrm{water},\;\mu_{w}$ (Pa·s)	1 × 10^−3^
$\mathrm{Density}\;\mathrm{of}\;\mathrm{Chlorella},\;\rho_{C}$ (kg/m^3^)	1050
Thickness of cell wall (μm)	0.1
$\mathrm{Electric}\;\mathrm{conductivity}\;\mathrm{of}\;\mathrm{cytoplasm},\;\sigma_{1}$(S/m)	0.5
$\mathrm{Electric}\;\mathrm{conductivity}\;\mathrm{of}\;\mathrm{cell}\;\mathrm{wall},\;\sigma_{2}$(S/m)	1 × 10^−8^
$\mathrm{Electric}\;\mathrm{conductivity}\;\mathrm{of}\;\mathrm{polystyrene}\;\mathrm{particle},\;\sigma_{3}$(S/m)	1 × 10^−16^

## Data Availability

Not applicable.

## References

[1] Wang Y., Zhao K., Tong N., Wang J. (2022). Separation of microalgae cells in a microfluidic chip based on AC Dielectrophoresis. J. Chem. Technol. Biotechnol..

[2] Iqbal H.M., Bilal M., Rasheed T., Ahmed I. (2017). High-value compounds from microalgae with industrial exploitability—A review. Front. Biosci..

[3] Liu B., Li D., Chen S., Wu N., Guan Y. (2021). Improving biological condition assessment accuracy by multimetric index approach with microalgae in streams and lakes. Sci. Total Environ..

[4] Vasistha S., Khanra A., Clifford M., Rai M. (2021). Current advances in microalgae harvesting and lipid extraction processes for improved biodiesel production: A review. Renew. Sustain. Energy Rev..

[5] Daneshvar E., Ok Y.S., Tavakoli S., Sarkar B., Shaheen S.M., Hong H., Luo Y., Rinklebe J., Song H., Bhatnagar A. (2021). Insights into upstream processing of microalgae: A review. Bioresour. Technol..

[6] Cao Q., Sun W., Yang T., Zhu Z., Jiang Y., Hu W., Wei W., Zhang Y., Yang H. (2022). The toxic effects of polystyrene microplastics on freshwater algae Chlorella pyrenoidosa depends on the different size of polystyrene microplastics. Chemosphere.

[7] Zhu Z.-L., Wang S.-C., Zhao F.-F., Wang S.-G., Liu F.-F., Liu G.-Z. (2019). Joint toxicity of microplastics with triclosan to marine microalgae Skeletonema costatum. Environ. Pollut..

[8] Zhao K., Wei Y., Dong J., Zhao P., Wang Y., Pan X., Wang J. (2022). Separation and characterization of microplastic and nanoplastic particles in marine environment. Environ. Pollut..

[9] Lagarde F., Olivier O., Zanella M., Daniel P., Hiard S., Caruso A. (2016). Microplastic interactions with freshwater microalgae: Hetero-aggregation and changes in plastic density appear strongly dependent on polymer type. Environ. Pollut..

[10] Li Z., Yi X., Zhou H., Chi T., Li W., Yang K. (2020). Combined effect of polystyrene microplastics and dibutyl phthalate on the microalgae Chlorella pyrenoidosa. Environ. Pollut..

[11] Abinandan S., Subashchandrabose S.R., Venkateswarlu K., Perera I.A., Megharaj M. (2019). Acid-tolerant microalgae can withstand higher concentrations of invasive cadmium and produce sustainable biomass and biodiesel at pH 3.5. Bioresour. Technol..

[12] Balaji S., Kalaivani T., Sushma B., Pillai C.V., Shalini M., Rajasekaran C. (2016). Characterization of sorption sites and differential stress response of microalgae isolates against tannery effluents from ranipet industrial area—An application towards phycoremediation. Int. J. Phytoremediation.

[13] Sajeesh P., Sen A.K. (2014). Particle separation and sorting in microfluidic devices: A review. Microfluid. Nanofluidics.

[14] Zhao K., Peng R., Li D. (2016). Separation of nanoparticles by a nano-orifice based DC-dielectrophoresis method in a pressure-driven flow. Nanoscale.

[15] Zhao K., Li D. (2017). Continuous separation of nanoparticles by type via localized DC-dielectrophoresis using asymmetric nano-orifice in pressure-driven flow. Sens. Actuators B Chem..

[16] Khoshmanesh K., Nahavandi S., Baratchi S., Mitchell A., Kalantar-Zadeh K. (2011). Dielectrophoretic platforms for bio-microfluidic systems. Biosens. Bioelectron..

[17] Zhang C., Khoshmanesh K., Mitchell A., Kalantar-Zadeh K. (2010). Dielectrophoresis for manipulation of micro/nano particles in microfluidic systems. Anal. Bioanal. Chem..

[18] Zhao K., Zhao P., Dong J., Wei Y., Chen B., Wang Y., Pan X., Wang J. (2022). Implementation of an Integrated Dielectrophoretic and Magnetophoretic Microfluidic Chip for CTC Isolation. Biosensors.

[19] Zhao K., Hu M., van Baalen C., Alvarez L., Isa L. (2023). Sorting of heterogeneous colloids by AC-dielectrophoretic forces in a microfluidic chip with asymmetric orifices. J. Colloid Interface Sci..

[20] Pysher M.D., Hayes M.A. (2007). Electrophoretic and Dielectrophoretic Field Gradient Technique for Separating Bioparticles. Anal. Chem..

[21] Kang Y., Cetin B., Wu Z., Li D. (2009). Continuous particle separation with localized AC-dielectrophoresis using embedded electrodes and an insulating hurdle. Electrochim. Acta.

[22] Zhao K., Li D. (2018). Tunable Droplet Manipulation and Characterization by ac-DEP. ACS Appl. Mater. Interfaces.

[23] Zhao K., Larasati L., Duncker B.P., Li D. (2019). Continuous Cell Characterization and Separation by Microfluidic Alternating Current Dielectrophoresis. Anal. Chem..

[24] Jiang T., Chen X., Ren Y., Tang D., Jiang H. (2021). Dielectric Characterization and Multistage Separation of Various Cells via Dielectrophoresis in a Bipolar Electrode Arrayed Device. Anal. Chem..

[25] Lapizco-Encinas B.H. (2019). On the Recent Developments of Insulator-Based Dielectrophoresis: A Review. Electrophoresis.

[26] Lapizco-Encinas B.H. (2020). Microscale electrokinetic assessments of proteins employing insulating structures. Curr. Opin. Chem. Eng..

[27] Perez-Gonzalez V.H., Gallo-Villanueva R.C., Cardenas-Benitez B., Martinez-Chapa S.O., Lapizco-Encinas B.H. (2018). Simple Approach to Reducing Particle Trapping Voltage in Insulator-Based Dielectrophoretic Systems. Anal. Chem..

[28] Zhao K., Li D. (2018). Manipulation and separation of oil droplets by using asymmetric nano-orifice induced DC dielectrophoretic method. J. Colloid Interface Sci..

[29] Zhao K., Li D. (2018). Direct current dielectrophoretic manipulation of the ionic liquid droplets in water. J. Chromatogr. A.

[30] Ramirez-Murillo C.J., de los Santos-Ramirez J.M., Perez-Gonzalez V.H. (2021). Toward Low-Voltage Dielectrophoresis-Based Microfluidic Systems: A Review. Electrophoresis.

[31] Tottori S., Misiunas K., Keyser U.F., Bonthuis D.J. (2019). Nonlinear Electrophoresis of Highly Charged Nonpolarizable Particles. Phys. Rev. Lett..

[32] Jones T.B. (1995). Electromechanics of Particles.

[33] Morgan H., Green N.G. (2003). AC Electrokinetics: Colloids and Nanoparticles.

[34] Zhang L., Zhu Y. (2010). Dielectrophoresis of Janus particles under high frequency ac-electric fields. Appl. Phys. Lett..

[35] Li D. (2013). Encyclopedia of Microfluidics and Nanofluidics.

[36] Nahavandi M. (2016). Continuous-Flow Separation of Malaria-Infected Human Erythrocytes Using DC Dielectrophoresis: An Electrokinetic Modeling and Simulation. Ind. Eng. Chem. Res..

[37] Das D., Biswas K., Das S. (2014). A microfluidic device for continuous manipulation of biological cells using dielectrophoresis. Med. Eng. Phys..

